# Supplementary figures and images for: Protective Effect of the Naringin–Chitooligosaccharide Complex on Lipopolysaccharide-Induced Systematic Inflammatory Response Syndrome Model in Mice
Source: Foods. 2024 Feb 14;13(4):576. doi: 10.3390/foods13040576 (PMC10887581; doi:10.3390/foods13040576)

## Slide 1
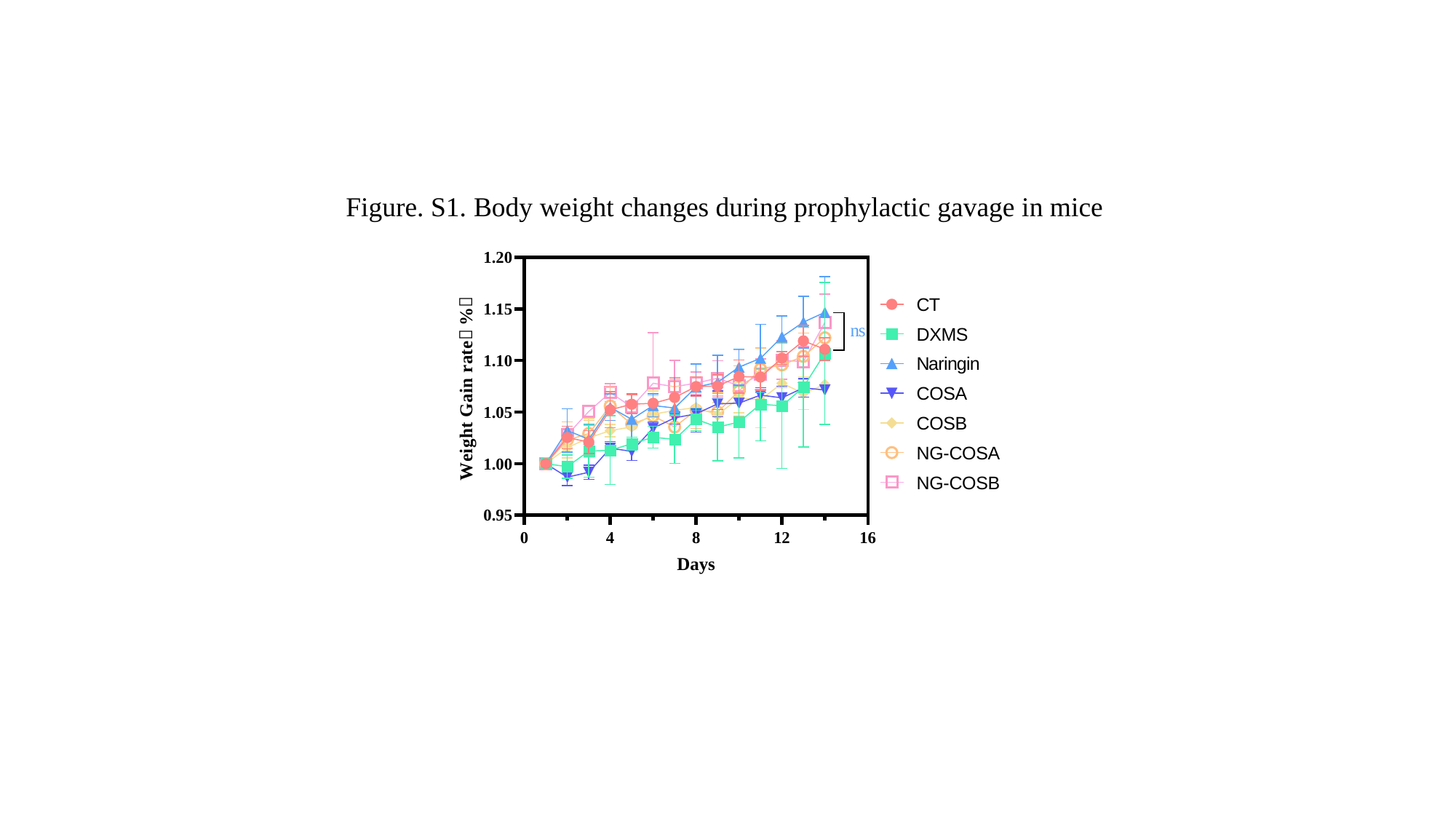

Figure. S1. Body weight changes during prophylactic gavage in mice

Supplement: Supplementary file 1 [file foods-13-00576-s001.zip › Figure S1.pptx]
